# Surgery Complicated by Self-Expandable Metallic Stents (SEMS) Tracheal Stent in a Congenital H-type Tracheo-Esophageal Fistula

**DOI:** 10.7759/cureus.22109

**Published:** 2022-02-10

**Authors:** Murugappan Nachiappan, Ravikiran Thota, Srikanth Gadiyaram

**Affiliations:** 1 Department of Surgical Gastroenterology and Minimally Invasive Surgery, Sahasra Hospitals, Bangalore, IND

**Keywords:** case report, tracheal sems, benign, congenital, tracheo-esophageal fistula

## Abstract

A congenital tracheo-esophageal fistula of the H-type is a rare variant. The diagnosis is usually missed because of mild symptoms. A long history of coughing during liquid intake and nocturnal cough may aid in the diagnosis. A delay in the diagnosis may have a deleterious effect on the lung because of recurrent infections. Surgery is the cornerstone of management. Self-expandable metallic stents (SEMS) do not have a role in the management of these fistulae. We report a case of a missed diagnosis of a congenital H-type fistula managed as an acquired tracheo-esophageal fistula with two attempts at conservative management with a tracheal self-expandable metallic stent. The difficulties and disadvantages of using self-expandable metallic stents for the management of benign tracheo-esophageal fistulae are also discussed.

## Introduction

Tracheo-esophageal fistula in adults is classified as either malignant or benign [1]. Malignant causes include advanced malignancies of the trachea or the esophagus. The most common benign causes include traumatic tracheal intubation, pressure necrosis caused by a long-standing tracheostomy tube cuff, and esophageal surgery, followed by inflammatory disorders such as tuberculosis and fungal infections. Congenital tracheo-esophageal fistula is one of the most common congenital anomalies in the neonatal age group. They are classified according to their anatomical configuration. The majority of congenital tracheo-esophageal fistulae are detected at neonatal age because of esophageal atresia. Congenital H-type tracheo-esophageal fistulae are present with mild symptoms and are the least prevalent type [2,3]. They may escape detection during the neonatal period to be present in adult life. Only about 20 cases of H-type fistulae have been recorded in English literature [4]. 

Long-term fistula closure rates with surgery for benign tracheo-esophageal fistula are over 90% [5]. Self-expandable metal stents have an established role in the treatment of malignant tracheo-esophageal fistulae [6,7]. Their role in benign tracheo-esophageal fistula is not well established. We describe a patient with a congenital H-type tracheo-esophageal fistula who was misdiagnosed earlier as an acquired one and was managed with a self-expandable metallic tracheal stent elsewhere. This case demonstrates the critical role of a thorough clinical history in etiological diagnosis as well as the technical difficulties posed by a previous tracheal SEMS during surgical repair of a tracheo-esophageal fistula. 

## Case presentation

**Figure 1:**
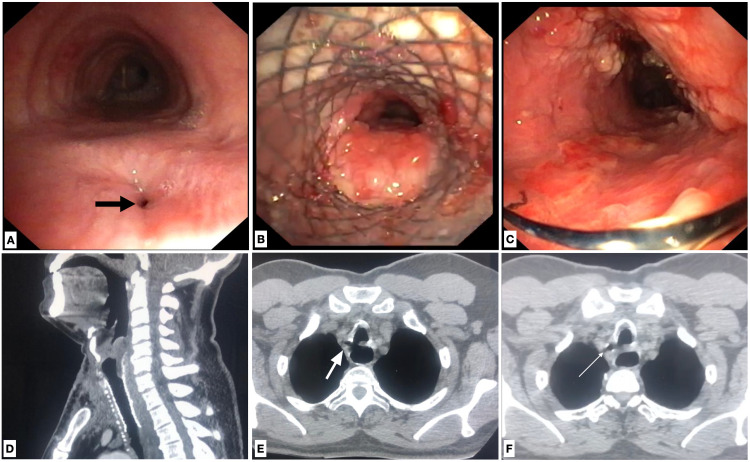
(A) Bronchoscopy showing the fistula (block arrow) in the membranous part of the trachea, (B) tracheal SEMS with granulation tissue, (C) post-stent removal granulation tissue in the trachea, (D) CT demonstrating the SEMS placement, (E) persistence of the fistula (block arrow) post-removal of SEMS, and (F) air foci in the tracheo-esophageal groove (line arrow) post-removal of SEMS. SEMS: self-expandable metallic stents.

**Figure 2:**
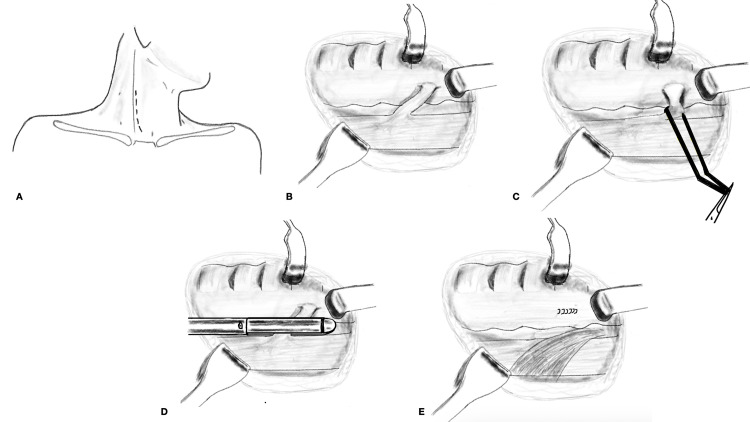
Illustration of surgical steps: (A) incision, (B) dissection in the tracheo-esophageal groove to expose the fistula, (C) looping of the fistula, (D) stapler division of the fistula, and (E) sternocleidomastoid interposition.

**Figure 3:**
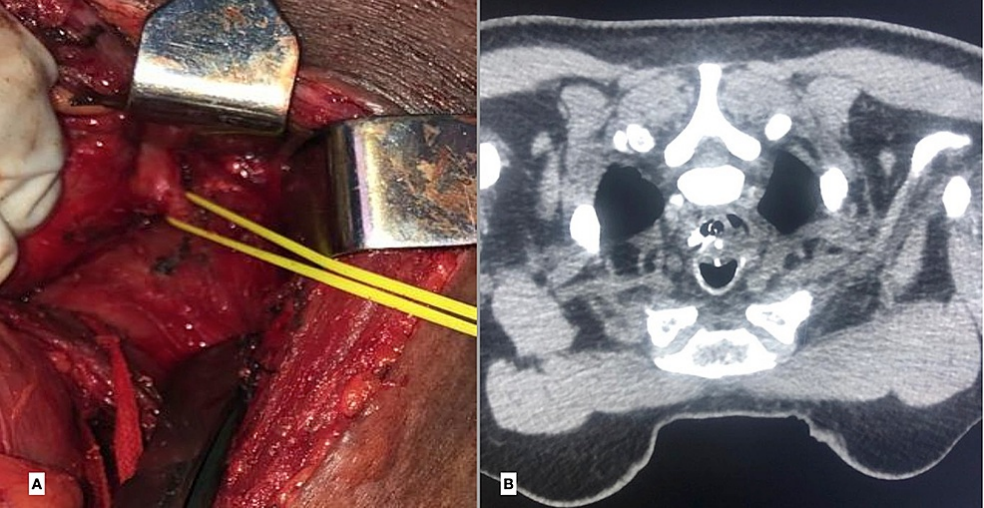
(A) Intra-operative photo showing the fistula looped with a vascular sling, (B) prone CT image post-repair showing the staples in trachea and the esophagus with buttressed sternocleidomastoid muscle in between the two with no evidence of contrast leak.

A 57-year-old male with no known medical conditions was referred to us for surgical management following the failure of endotherapy with a self-expandable metallic tracheal stent for an acquired tracheo-esophageal fistula. He had no previous history of cancer or surgical intervention. He had seen a pulmonologist seven months earlier for persistent cough with expectoration. During the evaluation, a tracheo-esophageal fistula 5 cm above the carina was found during flexible bronchoscopy (Figure 1A). An upper gastrointestinal endoscopy revealed a fistula on the esophageal side, approximately 20 cm from the incisors. High resolution computerised tomography (HRCT) of the thorax confirmed the fistula and showed a few subcentimetric lymph nodes adjacent to the fistula and lung infiltrates at the bases. A biopsy of the lymph nodes using endobronchial ultrasonography indicated mild inflammatory changes. The tracheo-esophageal fistula was diagnosed as acquired, and he had undergone a bronchoscopic placement of a self-expandable metallic tracheal stent along with antibiotic therapy and chest physiotherapy. He had a persistent cough at follow-up that required two bronchoscopic cauterization procedures of the granulation tissue, followed by a self-expandable metallic stent replacement for a persistent fistula three months after initial stent placement (Figure 1B-1D). A persistent tracheo-esophageal fistula was observed at bronchoscopy after the removal of the second stent. Subsequent to two unsuccessful fistula closure efforts with self-expandable metallic tracheal stents over a seven-month period, the patient was sent to us for surgical repair. A detailed enquiry revealed a history of nocturnal cough and bouts of coughing during eating since childhood. On clinical evaluation, the patient was hemodynamically stable and had bilateral basal lung crepitations. A HRCT thorax revealed a tracheo-esophageal fistula at the level of the upper border of the manubrium sterni and minimal extra-luminal air in the vicinity of the fistula, consistent with a sealed perforation (Figure 1E, 1F). The patient was diagnosed with a congenital H-type tracheo-esophageal fistula and was counseled for a surgical repair (Figure 2A-2E). After informed consent, the patient underwent an open surgical closure of the fistula through a right neck incision. There was extensive peri-tracheal fibrosis. The dissection was initially conducted in a virgin plane away from the fistula and then narrowed down toward it to avoid inadvertent breach of the fistula. A 5 mm fistula was found around 5 cm below the cricopharynx and just cranial to the level of the superior border of the manubrium sterni. It was about 1 cm long and coursing cranially from the esophagus toward the trachea (Figure 3A). After dissection, the fistula was looped and divided using an Endo GIATM 60 mm blue cartridge (Medtronic Inc, Minneapolis, MN, USA). A right sternocleidomastoid flap was inserted between the esophageal and tracheal staple lines. The patient's post-operative recovery was unremarkable. A post-operative CT with oral contrast done in a prone position showed no leakage of the contrast (Figure 3B). At two years' follow-up after surgery, he remains asymptomatic.

## Discussion

Among congenital tracheo-esophageal fistulae, the H-type is the least prevalent. Typically, these congenital fistulae are placed approximately 19-20 cm from the incisors. These fistulae follow an oblique path, with the tracheal end being cephalad in relation to the esophageal end, most likely due to differential growth [8]. This also accounts for the absence of food and liquids in the sputum. Delayed presentations, like in our case, may occur as a result of the patient's disregard for the initial mild symptoms. These patients may have a history of coughing with liquid intake and nocturnal coughing dating all the way back to childhood. The value of a thorough clinical history cannot be overstated, and history from childhood would almost certainly indicate a congenital cause. Delay in diagnosis may result in repeated aspiration, pulmonary infections, and unnecessary procedures in some. Thus, in a patient with cough following intake of liquids, recurrent chest infections, and nocturnal cough since childhood, a congenital H-type tracheo-esophageal fistula must be considered in the differential diagnosis. An early diagnosis, therefore, requires a high index of suspicion. They are frequently missed during upper gastrointestinal endoscopy because of their anterior location. Bronchoscopy can be used to determine the location of a fistula in the membranous trachea. A barium swallow in the prone position or a CT with oral contrast may be necessary to confirm the diagnosis. An MRI could also demonstrate the fistula.

The management option for a congenital fistula is by an open surgical resection that can be achieved through neck exploration in the majority of cases. Video-assisted thoracoscopic repair when the fistula is lower down has been described. Other options for the management of these fistulae include endoscopic repair and patch closure similar to that used in atrial septal defect repair [9]. 

Many patients experience troublesome symptoms following tracheal SEMS deployment. In one study, sore throats, hemoptysis, and productive cough were seen in 21%, 15%, and 25% of the patients, respectively [10]. Distressing complications include stent migration, granulation tissue ingrowth, and recurrent stenoses, seen in 6%, 34%, and 18% of patients, respectively [10,11]. Once deployed, these stents can be challenging to remove and may cause stent fracture, perforation, or mucosal injury. In 2005, the FDA issued an advisory against the use of self-expandable metallic stents for benign airway disease in view of an increase in the number of adverse events. Further, it was urged that management decisions for such disorders should be discussed in a multidisciplinary team, including surgeons [12]. There is no role for self-expandable metallic stents in the congenital tracheo-esophageal fistula. In our case, imaging revealed a sealed perforation that was evidenced by extensive peritracheal inflammation, which complicated the dissection during surgery. The risk of inadvertent recurrent laryngeal nerve injury is likely to be higher in such situations. 

The principles of open surgery include determining the side of the neck to explore based on the orientation of the fistula (determined on preoperative imaging), looping of the fistula following adequate dissection, tensionless repair on the membranous trachea side, and interposition of vascularized tissue between the suture/staple lines to prevent recurrence.

## Conclusions

A history of cough following food intake from childhood should lead to a high index of suspicion of a congenital H-type tracheo-esophageal fistula. Surgery is the only curative option. A self-expandable metallic stent has no role in the management of this fistula. Once deployed, they increase the difficulty of subsequent surgery.
